# Language Differences Among Individuals with Normal Cognition, Amnestic and Non-Amnestic MCI, and Alzheimer’s Disease

**DOI:** 10.1093/arclin/acac080

**Published:** 2022-10-15

**Authors:** Ioannis Liampas, Vasiliki Folia, Renia Morfakidou, Vasileios Siokas, Mary Yannakoulia, Paraskevi Sakka, Nikolaos Scarmeas, Georgios Hadjigeorgiou, Efthimios Dardiotis, Mary H Kosmidis

**Affiliations:** Department of Neurology, University Hospital of Larissa, School of Medicine, University of Thessaly, Larissa, Greece; Lab of Cognitive Neuroscience, School of Psychology, Aristotle University of Thessaloniki, Greece; Lab of Cognitive Neuroscience, School of Psychology, Aristotle University of Thessaloniki, Greece; Department of Neurology, University Hospital of Larissa, School of Medicine, University of Thessaly, Larissa, Greece; Department of Nutrition and Dietetics, Harokopio University, Athens, Greece; Association of Alzheimer's Disease and Related Disorders, Marousi, Athens, Greece; First Department of Neurology, Aeginition Hospital, National and Kapodistrian University of Athens Medical School, Athens, Greece; Taub Institute for Research in Alzheimer's Disease and the Aging Brain, The Gertrude H. Sergievsky Center, Department of Neurology, Columbia University, New York, USA; Department of Neurology, University Hospital of Larissa, School of Medicine, University of Thessaly, Larissa, Greece; Department of Neurology, Medical School, University of Cyprus, Nicosia, Cyprus; Department of Neurology, University Hospital of Larissa, School of Medicine, University of Thessaly, Larissa, Greece; Lab of Cognitive Neuroscience, School of Psychology, Aristotle University of Thessaloniki, Greece

**Keywords:** fluency (verbal/nonverbal), amnesia, Alzheimer’s disease, language and language disorders, mild cognitive impairment

## Abstract

**Objective:**

To investigate differences in language performance among older adults with normal cognition (CN), mild cognitive impairment (MCI), and Alzheimer’s disease (ad). Owing to the conflicting literature concerning MCI, discrepancies between amnestic (aMCI) and non-amnestic MCI (naMCI) were explored in greater detail.

**Method:**

The study sample was drawn from the older (>64 years) HELIAD cohort. Language performance was assessed via semantic and phonemic fluency, confrontation naming, verbal comprehension, verbal repetition as well as a composite language index. Age, sex, and education adjusted general linear models were used to quantify potential pairwise differences in language performance.

**Results:**

The present analysis involved 1607 participants with CN, 146 with aMCI [46 single and 100 multi-domain aMCI], 92 with naMCI [41 single and 51 multi-domain naMCI], and 79 with ad. The mean age and education of our predominantly female (60%) participants were 73.82 (±5.43) and 7.98 (±4.93) years, respectively. MCI individuals performed between those with CN and ad, whereas participants with aMCI performed worse compared to those with naMCI, especially in the semantic fluency and verbal comprehension tasks. Discrepancies between the aMCI and naMCI groups were driven by the exquisitely poor performance of multi-domain aMCI subgroup.

**Conclusions:**

Overall, individuals could be hierarchically arranged in a continuum of language impairment with the CN individuals constituting the healthy reference and naMCI, aMCI, ad patients representing gradually declining classes in terms of language performance. Exploration of language performance via separation of single from multi-domain naMCI provided a potential explanation for the conflicting evidence of previous research.

## Introduction

Language performance has been long considered as a potential screening tool in the differentiation of normal cognition (CN) from mild cognitive impairment (MCI) and dementia, especially of the Alzheimer’s (ad) type (McDonnell et al., 2020; Vaughan, Coen, Kenny, & Lawlor, 2018; Wakefield, McGeown, Shanks, & Venneri, 2014). Language impairment and most notably semantic verbal fluency (SVF) impairment becomes more prominent as global cognitive impairment progresses from CN to MCI and subsequently to dementia (Teng et al., 2013; Weakley, Schmitter-Edgecombe, & Anderson, 2013). Previous research, however, predominantly focused on amnestic MCI (aMCI), with only limited and occasionally conflicting reports assessing the non-amnestic (naMCI) subtype.

Published studies emphasizing semantic and phonemic verbal fluency (PVF) have occasionally corroborated that patients with amnestic and naMCI perform similarly on both tasks (Rinehardt et al., 2014); however, there is also a considerable amount of evidence suggesting otherwise. A number of researchers have found that individuals with naMCI outperform those with aMCI in terms of SVF but not PVF (Kim, Kim, & Kim, 2019; Teng et al., 2013). On the other hand, several studies have demonstrated that older adults with naMCI may perform worse on semantic or PVF tasks compared to those with aMCI (Brandt & Manning, 2009; Weakley et al., 2013). These discrepancies among studies appear to be at least partially driven by the proportion of individuals with single (aMCI-SD) and multidomain aMCI (aMCI-MD) (Brandt & Manning, 2009; Weakley et al., 2013). Previous studies, however, did not account for the potential variation introduced by the different numbers of participants with single (naMCI-SD) and multidomain naMCI (naMCI-MD). Apart from verbal fluency, confrontation naming has been also investigated (although less extensively), with several reports indicating a worse performance in the aMCI group (Kim et al., 2019), while others suggesting that only the aMCI-MD subtype (and not aMCI-SD) is truly affected in terms of confrontation naming (Weakley et al., 2013).

Overall, despite the uniformly accepted notion that MCI lies on the CN-dementia continuum of language decline, substantial between-study discrepancies have emerged among studies investigating the amnestic and non-amnestic subtypes of MCI. Neuropathological and imaging investigations, on the other hand, have generated several consistently reproduced disparities between the two groups. Evidence suggests that individuals with naMCI have higher densities of Lewy bodies (LBs) in the brain and more widespread disruption of white matter microstructure while exhibiting decreased prefrontal metabolism, significant prefrontal cortex atrophy and basal forebrain grey matter loss as well as greater clinicopathological affinity with Parkinson’s disease (PD) (Coutinho et al., 2015; Dugger et al., 2015; Rojas & Bettcher, 2016). Patients with aMCI, on the other hand, present a greater propensity for neurofibrillary tangles within the temporal and parietal lobes, more prominent hippocampal, entorhinal and amygdala atrophy, as well as lower levels of amyloid-β in the cerebrospinal fluid (Coutinho et al., 2015; Csukly et al., 2016; Dugger et al., 2015; Qin et al., 2020). These neuropathological and structural disparities appear to be on the same tract with the heterogeneous follow-up courses of aMCI and naMCI; that is the higher incidence of ad in the aMCI group (Espinosa et al., 2013; Fischer et al., 2007; Peltz, Corrada, Berlau, & Kawas, 2011), the greater incidence of PD, DLB (dementia with LBs), and FTLD (frontotemporal lobe degeneration) in the naMCI group and the affinity of both multidomain entities with vascular and mixed dementia (depending on the relative involvement of the neurovascular unit) (Bombois et al., 2008; Ferman et al., 2013; Yarnall, Rochester, & Burn, 2013).

Taking into account the different neuropsychological profiles of the above-listed neurodegenerative entities (roughly: greater attention and executive decline in DLB, PD, and FTLD and more prominent episodic memory decline in ad), it is possible that their “MCI equivalents” might share analogous neuropsychological disparities (Blanc, 2013; Dalrymple-Alford, 2001; Hamilton et al., 2021; Sadiq et al., 2017). Language performance is quite complex in the number of cognitive functions needed to perform it, with verbal fluency tasks being heavily based on both executive skills and semantic memory stores, whereas naming, comprehension, and repetition tasks rely on the semantic reservoir as well as perceptual skills (Amunts, Camilleri, Eickhoff, Heim, & Weis, 2020; Brouillette et al., 2011; Hodges, Salmon, & Butters, 1990; Kavé & Sapir-Yogev, 2020; Rende, Ramsberger, & Miyake, 2002; Rohrer et al., 2007). Based on the heterogeneous cognitive skills required for the different components of language, it is probable that language subdomains will differentially decline in amnestic and naMCI, with aMCI individuals exhibiting greater defects in primarily memory-dependent language tasks.

Therefore, in view of the aforementioned literature inconsistencies as well as the remarkably limited investigation of language indices other than verbal fluency, we investigated the language differences among older individuals with CN, MCI, and ad, focusing on the potential discrepancies between patients with amnestic and naMCI. By exploring language differences among individuals with aMCI-SD, naMCI-SD, aMCI-MD, and naMCI-MD, we specifically sought to address the major literature gap on the differential effect of naMCI-MD pathology on language impairment and potentially explain the contradictory findings of previous papers (Nutterupham et al., 2008; Weakley et al., 2013). Distinguishing language abilities (and possibly various other neuropsychological domains) among the MCI subgroups would greatly enhance our understanding of the neuropsychological and by extension neuropathological correlates of each MCI subtype and assist in the early and more accurate identification of cognitively impaired individuals. Of note, language has been proposed to provide comparable results or even outcompete episodic memory in the early diagnosis and prediction of incident MCI (even amnestic) in CN older adults (McCullough, Bayles, & Bouldin, 2019; Oulhaj, Wilcock, Smith, & de Jager, 2009). Similarly, language assessment has been suggested to aid in the earlier and more sensitive identification of prodromal and early stages of ad (Sherman, Henderson Jr., Flynn, Gair, & Lust, 2021; Szatloczki, Hoffmann, Vincze, Kalman, & Pakaski, 2015). Overall, the present attempt is important since research so far has addressed the issue of clinical and prognostic heterogeneity of the broad MCI concept (Forlenza et al., 2008; Hughes, Snitz, & Ganguli, 2011). First, subtyping MCI will delineate subgroups that are more clinically homogeneous, thus enhancing utility and validity of diagnosis (Kendell, et al., 2003). Distinguishing between aMCI and naMCI is a step towards this direction and shows growing ecological validity; however, further integrative research is needed to justify this distinction (Kendell et al., 2003). Therefore, distinguishing language performance measures within MCI subtypes will add to the diagnostic specificity and lead to greater predictive value of the diagnosis. The clinical benefit of such an attempt is the facilitation of treatment and management options that are more specific to the patients’ condition.

## Methods

The objective of the present study was to determine the existence of disparities in the language ability of older individuals with different cognitive backgrounds, i.e., CN, aMCI, naMCI, and ad, as well as to quantify pairwise differences. Reporting was performed according to the Strengthening the Reporting of Observational Studies in Epidemiology (STROBE) recommendations (von Elm et al., 2014). Study procedures were approved by the Institutional Ethics Review Boards of the University of Thessaly and the National and Kapodistrian University of Athens. All participants or surrogates had provided informed consent, prior to participation.

The study sample was drawn from the population-based Hellenic Longitudinal Investigation of Aging and Diet (HELIAD) study. The design and rationale of the HELIAD cohort have been published previously in more detail (Dardiotis, Kosmidis, Yannakoulia, Hadjigeorgiou, & Scarmeas, 2014; Liampas et al., 2022). Briefly, the HELIAD study explores the epidemiology of dementia, cognitive impairment, as well as other neuropsychological disorders in the aging Greek population. Participants were selected through random sampling from among the elderly (>64 years) rosters of two Greek municipalities, Marousi (Athens, Southern Greece), and Larissa (Thessaly, Central Greece). Extensive (~2–2.5 hours long), multidisciplinary evaluations involving structured interviews, administration of validated questionnaires, physical examinations, laboratory investigations, and neuropsychological assessments were performed by a collaboration of certified neurologists, trained neuropsychologists, and dieticians. If participants were unable to provide relevant information, we resorted to their carers (spouse, first degree relatives, etc.). The present study was based on the baseline evaluations of the HELIAD cohort, conducted between 2009 and 2015. Study assessments relevant to the present analysis are described below in greater detail.

## Neuropsychological Assessments & Diagnostic Procedures

A comprehensive neuropsychological evaluation was performed by trained neuropsychologists: Global cognition and Orientation (MMSE) (Folstein, Folstein, & McHugh, 1975), Non-verbal and Verbal Memory (Medical College of Georgia—MCG—Complex Figure Test (Lezak, 2004); Greek Verbal Learning Test (Vlahou et al., 2013)), Language (semantic and phonological verbal fluency (Kosmidis, Vlahou, Panagiotaki, & Kiosseoglou, 2004); subtests of the Greek version of the Boston Diagnostic Aphasia Examination short form, namely, the Boston Naming Test-short form, and selected items from the Complex Ideational Material Subtest, to assess verbal comprehension and repetition of words and phrases (Tsapkini, Vlahou, & Potagas, 2010)), visuospatial ability (Judgment of Line Orientation (Benton, 1994; Kosmidis, Tsotsi, Karambela, Takou, & Vlahou, 2010) abbreviated form; MCG Complex Figure Test copy condition, Clock Drawing Test (Bozikas, Giazkoulidou, Hatzigeorgiadou, Karavatos, & Kosmidis, 2008)), attention and information processing speed (trail making test—TMT—(Vlahou & Kosmidis, 2002) Part A), executive functioning (TMT Part B; Anomalous sentence repetition; graphical sequence test; motor programming (Lezak, Howieson, Loring, Hannay, & Fischer, 2004); months forwards and backwards), and a gross estimate of Intellectual level (a Greek multiple choice vocabulary test (Giaglis, Kyriazidou, Paraskevopoulou, Tascos, & Kosmidis, 2010)) were administered. Grouping of the aforementioned functions was performed according to an a priori neuropsychological knowledge of the particular cognitive domains reflected by each test (Bougea et al., 2019).

Regarding language assessments in specific, for the purposes of the present study SVF and PVF were evaluated according to a word fluency test consisting of two parts. Individuals were first asked to generate as many different words as possible, belonging to one semantic category (objects), whereas, in the second part, participants were asked to generate as many different words as possible, beginning with one Greek letter [a] (alpha). Participants were instructed to immediately begin generating items, following the announcement of the category or letter, and each trial lasted for 60 seconds. Regarding word search and production, no instructions were given, to ensure that any cognitive strategies would be spontaneously employed by the examinees. Participants were told to abstain from reporting proper nouns (regarding the phonemic test), as well as repetitions and word variations. Finally, confrontation naming was evaluated based on the 15-item Boston Naming Test-short form (BNT-sf) while verbal comprehension and repetition of words and phrases were assessed using selected items from the Complex Ideational Material Subtest (12 and 6-item tests respectively) (Folia et al., 2022).

To assess language overall, a composite language index was derived from all the above listed variables: the number of words generated in the SVF and PVF tasks, as well as the number of correct responses in the BNT-sf, verbal comprehension, and repetition tasks. To accomplish this, individual test raw scores were converted into *z*-scores using mean and standard deviation values of the CN group of individuals at baseline. Individual test *z*-scores were in turn averaged to generate a composite *z*-score for the cognitive function of language.

The diagnostic classification of the participants according to their cognitive status was established during expert consensus meetings involving senior neurologists and neuropsychologists. Dementia and ad were diagnosed according to the Diagnostic and Statistical Manual of Mental Disorders-IV-text revision criteria (American Psychiatric Association, 2000) and the National Institute of Neurological and Communicative Disorders and Stroke/Alzheimer Disease and Related Disorders Association criteria (McKhann et al., 1984), respectively. MCI and its subtypes were diagnosed according to the Petersen criteria (Petersen, 2004). MCI was categorized as amnestic in cases with isolated amnestic impairment (aMCI-SD) or multi-domain impairment involving the function of memory (aMCI-MD), and as non-amnestic in cases with isolated language, attention, executive, or visuo-perceptual impairment (naMCI-SD) or any combination of the above listed impairments not involving memory (naMCI-MD).

A detailed description of the implemented diagnostic approach is provided elsewhere (Kosmidis et al., 2018; Vlachos et al., 2020). Briefly, all participants underwent a standard physical and neurological examination. Particular focus was placed on identifying potential comorbidities that could affect cognitive performance through screening the participants for depression (Geriatric Depression Scale), anxiety (Anxiety Subscale of the Hospital Anxiety and Depression Scale), behavioral symptoms (Neuropsychiatric Inventory scale and the Columbia University Scale for Psychopathology in Alzheimer’s Disease), Parkinson disease, essential tremor, and dementia with Lewy bodies (DLB). In order to determine the coexistence of vascular disorder, we collected information about a past diagnosis of stroke or transient ischemic attack (TIA), or a history of neurological symptoms consistent with stroke or TIA. When there was a positive history, further information on the date of onset, duration, constellation of symptoms, admission to hospital, or any kind of rehabilitation treatment was elicited. In addition, we followed up reports of memory deterioration with questions regarding its onset (e.g., sudden loss of cognitive function concurrent with a stroke, a fluctuating, or stepwise progression of their memory difficulties). Comorbid conditions were evaluated according to a combined approach, gathering information from self-reported and/or carer-reported history, current medications, and medical test results, when available. Further query involved information on participants’ mental, physical, and social activities, sleep and dietary habits, subjective memory, or other cognitive complaints, as well as their ability to perform daily tasks.

## Outcome measures & statistical analysis

Differences in the composite language index, SVF, PVF, confrontation naming, verbal comprehension, as well as repetition, among (and between) individuals with different cognitive status were defined as the primary outcomes of interest. Gender was treated as a categorical variable, while age in years and years of formal education were treated as scale variables. Adjusted univariate general linear models (GLMs) were used to explore and quantify the presence of differences in the composite, as well as the five primary language indices, among (and between) the different cognitive classes (CN, aMCI, naMCI, and ad). Univariate GLM provides regression analysis and analysis of variance for dependent variables by several factors and covariates. Language measures were sequentially inserted as the dependent parameters into separate GLMs, while each analysis was adjusted for the factor of sex, as well as the covariates of age and years of education. Post-hoc between group comparisons (4 groups, i.e., 6 between group comparisons per language measurement, *p*-values were adapted according to the Bonferroni correction) were provided separately.

Language differences were additionally investigated among single and multi-domain amnestic and naMCI (main focus of the current research). In this way, we examined the potentially differential impact of multi-domain pathology on language impairment. Language indices were analyzed using an interchangeable statistical approach as described above (univariate GLMs corrected according to the Bonferroni correction and adjusted for age, sex and years of formal education).

All statistical analyses were performed using the IBM SPSS Statistics Software Version 25 (Chicago, IL, USA). The conventional threshold of 5% was implemented for the revelation of statistical significance.

## Results

### Baseline characteristics and missing data

The HELIAD study included 1984 participants at baseline. Among them, 36 participants were excluded due to missing data, leading to an inconclusive cognitive diagnosis, and 24 were excluded owing to the identification of other dementias (9 individuals were diagnosed with vascular dementia, 8 with Parkinson’ s Disease or Dementia with Lewy Bodies, and 7 with less common entities). The remaining 1924 participants were classified as follows: 1607 with CN, 146 with aMCI, 92 with naMCI and 79 with AD. Baseline demographic information and neuropsychological measurements are descriptively presented per cognitive group in Table 1. The mean age of our sample was 73.82 years (SD = 5.43), ranging between 65 and 99 years. Participants were predominantly women: 779 men (40.5%) and 1145 women (49.5%). The mean duration of formal education (in years) was 7.98 years (SD = 4.93), ranging between 0 and 21 years of education. Among MCI participants, there were 46 with aMCI-SD, 100 with aMCI-MD, 41 with naMCI-SD and 51 with naMCI-MD. Extensive details with respect to the baseline characteristics of these participants are provided elsewhere (Vlachos et al., 2020).

**Table 1:** Baseline characteristics of CN, aMCI, naMCI, and AD individuals

Variable	CN	aMCI	naMCI	AD
Age in years (*N* = 1924)	73.31 ± 5.19 (65.03–91.46)	74.84 ± 5.20 (65.12–86.78)	75.91 ± 5.26 (65.17–89.04)	79.96 ± 6.36 (65.25–99.65)
Gender (M/F) (*N* = 1924)	643 / 964 (40.0/60.0%)	65 / 81 (45.5/55.5%)	39 / 53 (42.4/57.6%)	32 / 47 (40.5/59.5%)
Years of Education (*N* = 1923)	8.17 ± 4.86 (0–21)	7.20 ± 5.44 (0–21)	6.92 ± 4.78 (0–17)	6.87 ± 5.30 (0–18)
Composite language index (*N* = 1884)	−0.03 ± 0.081 (−3.43–1.17)	−0.76 ± 1.04 (−4.06–1.11)	−0.65 ± 0.95 (−3.18–1.08)	−1.41 ± 1.31 (−4.64–1.06)
SVF performance (*N* = 1873)	15.70 ± 4.98 (0–33)	11.30 ± 5.12 (1–27)	13.58 ± 5.48 (4–28)	7.77 ± 4.73 (0–21)
PVF performance (*N* = 1825)	7.39 ± 4.52 (0–24)	4.92 ± 4.14 (0–21)	5.72 ± 3.67 (0–21)	3.57 ± 3.69 (0–14)
BNT-sf (*N* = 1874)	10.88 ± 2.88 (0–15)	8.92 ± 3.13 (2–15)	9.29 ± 2.97 (2–15)	7.21 ± 4.03 (1–15)
Comprehension (*N* = 1822)	10.88 ± 1.47 (1–12)	9.56 ± 2.09 (2–12)	10.07 ± 1.83 (5–12)	8.16 ± 2.82 (2–12)
Repetition (*N* = 1869)	4.78 ± 1.29 (0–6)	3.94 ± 1.62 (0–6)	3.90 ± 1.50 (1–6)	3.37 ± 1.68 (0–6)
Global Cognition (*N* = 1889)	−0.07 ± 0.71 (−3.72–1.37)	−0.94 ± 0.86 (−3.44–0.67)	−0.70 ± 0.77 (−2.63–1.03)	−1.91 ± 1.22 (−5.14–0.82)
Composite Memory Index (*N* = 1867)	−0.04 ± 0.84 (−3.35–2.17)	−1.29 ± 0.63 (−2.63–0.48)	−0.54 ± 0.73 (−2.05–1.64)	−1.80 ± 0.66 (−2.71–1.62)
Composite Executive Function Index (*N* = 1880)	−0.07 ± 0.73 (−3.52–1.96)	−0.79 ± 0.88 (−3.25–0.90)	−0.70 ± 0.83 (−3.02–0.92)	−1.60 ± 1.39 (−5.63–0.68)
Composite Attention-Speed Index (*N* = 1770)	−0.10 ± 1.02 (−6.58–1.46)	−1.06 ± 1.59 (−6.66–1.60)	−0.93 ± 1.25 (−6.66–0.95)	−2.50 ± 2.48 (−6.66–0.86)
Composite Visuospatial Ability Index (*N* = 1855)	−0.04 ± 0.89 (−7.08–1.76)	−0.69 ± 1.27 (−6.07–1.19)	−0.66 ± 1.06 (−3.42–1.19)	−1.88 ± 2.04 (−6.96–0.98)

*Notes:* CN: normal cognition; aMCI: amnestic mild cognitive impairment; naMCI: non-amnestic MCI; AD: Alzheimer’ s disease; *N*: number of participants with available data per variable; M/F: male/female; SVF: semantic verbal fluency; PVF: phonemic verbal fluency; BNT-sf: Boston Naming Test short form.

### Language differences among older individuals based on their cognitive status

Table 2 contains a detailed illustration of the language differences among (and between) Greek older individuals with dissimilar cognitive backgrounds, adjusted to the average effect of sex, the mean age and education of our sample. Overall, we could hierarchically arrange our participants in a hypothetical continuum of language decline, with CN individuals constituting the healthy reference and naMCI, aMCI and AD patients representing gradually declining groups in terms of language performance. A more detailed description of the pattern of language differences is provided below.

**Table 2:** Differences in language performance (with 95% Confidence Intervals and corresponding *p*-values) among individuals with CN, aMCI, naMCI, and AD, adjusted to the mean age and formal education of our sample as well as to the average effect of sex

Language variable	CN vs. aMCI	CN vs. naMCI	CN vs.AD	aMCI vs. naMCI	aMCI vs. AD	naMCI vs. AD
Composite Language Index	**0.56 (0.41, 0.70), <0.001**	**0.39 (0.21, 0.57), <0.001**	**1.01 (0.81, 1.21), <0.001**	−0.17 (−0.39, 0.05), 0.258	**0.45 (0.21, 0.69), <0.001**	**0.62 (0.36, 0.88), <0.001**
SVF performance	**3.70 (2.65, 4.75), <0.001**	1.15 (−0.16, 2.45), 0.123	**6.06 (4.53, 7.58), <0.001**	**−2.55 (−4.16, −0.95), <0.001**	**2.36 (0.57, 4.14), 0.003**	**4.91 (2.98, 6.85), <0.001**
PVF performance	**1.91 (1.05, 2.77), <0.001**	0.82 (−0.23, 1.88), 0.235	**2.78 (1.54, 4.01), <0.001**	−1.09 (−2.39, 0.22), 0.170	0.87 (−0.58, 2.32), 0.684	**1.96 (0.39, 3.52), 0.006**
Naming	**1.42 (0.91, 1.93), <0.001**	**0.86 (0.22, 1.50), 0.002**	**2.49 (1.75, 3.23), <0.001**	−0.56 (−1.35, 0.23), 0.360	**1.07 (0.20, 1.94), 0.007**	**1.63 (0.69, 2.57), <0.001**
Comprehension	**1.15 (0.80, 1.49), <0.001**	**0.55 (0.13, 0.97), 0.004**	**2.39 (1.89, 2.90), <0.001**	**−0.59 (−1.12, −0.07), 0.017**	**1.25 (0.66, 1.84), <0.001**	**1.84 (1.21, 2.48), <0.001**
Repetition	**0.65 (0.39, 0.91), <0.001**	**0.60 (0.27, 0.92), <0.001**	**0.99 (0.60, 1.37), <0.001**	−0.05 (−0.45, 0.35), 1.000	0.34 (−0.11, 0.79), 0.269	0.39 (−0,09, 0.87), 0.195

*Notes:* CN: normal cognition; aMCI: amnestic mild cognitive impairment; naMCI: non-amnestic MCI; AD: Alzheimer’ s disease; SVF: semantic verbal fluency; PVF: phonemic verbal fluency; BNT-sf: Boston Naming Test short form; Positive mean differences conventionally represent a superior performance of the first group of participants as quoted at the top of each column, while negative mean differences correspond to a superior performance of the second group of participants as quoted at the top of each column; **bold** denotes statistical significance of the results.

**Fig. 1:**
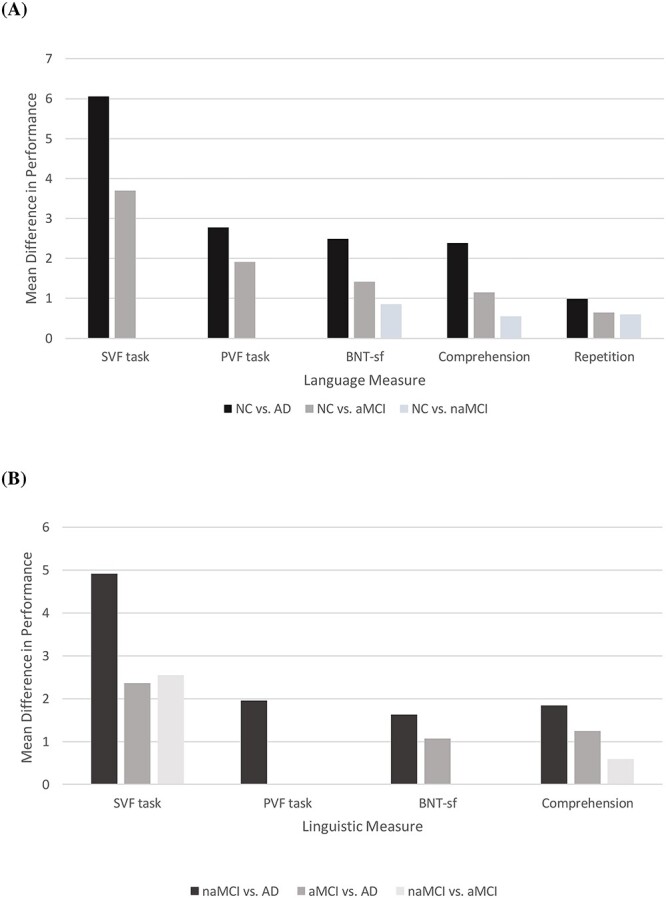
Schematic illustration of the significant adjusted differences **(A)** between individuals with and without CN and **(B)** among individuals without CN, in the main language measures. Differences conventionally represent a superior score of the first group of participants as quoted in the note clarifying the color coding of each column at the bottom of each figure. CN: normal cognition; aMCI: amnestic mild cognitive impairment; naMCI: non-amnestic MCI; AD: Alzheimer’ s disease; SVF: semantic verbal fluency; PVF: phonemic verbal fluency; BNT-sf: Boston Naming Test short form.

Compared to the healthy reference (CN), participants with AD and aMCI had lower scores in both composite and individual language indices: differences between the CN and AD groups were more prominent (than between the CN and aMCI groups). In contrast, patients with naMCI performed similar to individuals with CN on the SVF and PVF tasks. At the same time, differences in the composite language index, confrontation naming, verbal comprehension, and repetition were less prominent between the CN and naMCI groups, that between the CN and aMCI groups (Figure 1a). Regarding the AD group, individuals with dementia consistently performed worse than those with naMCI, with the only exception of verbal repetition (similar performance). However, smaller-sized disparities were determined compared to those with aMCI in the composite language index, SVF, BNT-sf, and verbal comprehension, whereas the aMCI and AD groups documented similar scores in both PVF and verbal repetition (Figure 1b). Finally, naMCI patients outperformed those with aMCI in the SVF and comprehension tasks. While the naMCI group consistently recorded higher average scores than the aMCI group, these differences did not reach the statistical threshold of significance in the remaining language variables (Figure 1b).

### Language differences among older individuals with different MCI subtypes

**Fig. 2:**
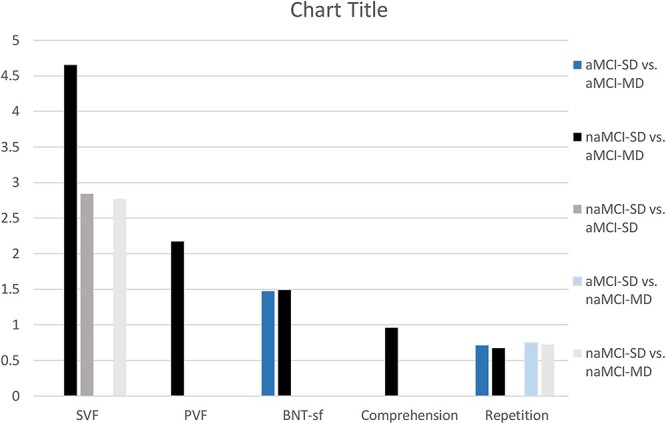
Schematic illustration of the significant adjusted differences in the main language variables among individuals with aMCI-SD, aMCI-MD, naMCI-SD, and naMCI-MD. Differences conventionally represent a superior score of the first group of participants as quoted in the note clarifying the color coding of each column, at the right side of the graph. MCI: mild cognitive impairment; aMCI-SD: single-domain amnestic MCI; naMCI-SD: single-domain non-amnestic MCI; aMCI-MD: multi-domain amnestic MCI; naMCI-MD: multi-domain non-amnestic MCI SVF: semantic verbal fluency; PVF: phonemic verbal fluency; BNT-sf: Boston Naming Test short form.

Language differences among the single and multi-domain amnestic and naMCI groups were in general indicative of more prominent language impairments in the aMCI-MD group relative to the other MCI categories (Table 3) and (Figure 2). In specific, individuals with aMCI-MD performed worse than those with naMCI-SD in both all (composite and individual) language measures. Compared to individuals with aMCI-SD, those with aMCI-MD recorded lower composite language as well as BNT-sf and repetition scores, whereas no differences were observed in comparison with the naMCI-MD group. Additionally, participants with naMCI-SD outperformed those with naMCI-MD in the SVF and repetition tasks. Finally, the aMCI-SD group performed worse than the naMCI-SD group in the SVF test.

**Table 3:** Differences in language performance (with 95% Confidence Intervals and corresponding *p*-values) among individuals with aMCI-SD, aMCI-MD, naMCI-SD, and naMCI-MD, adjusted to the mean age and formal education of our sample as well as to the average effect of sex

Language variable	aMCI-MD vs. aMCI-SD	aMCI-MD vs. naMCI-SD	aMCI-MD vs. naMCI-MD	aMCI-SD vs. naMCI-SD	aMCI-SD vs. naMCI-MD	naMCI-MD vs. naMCI-SD
Composite language index	**−0.45 (−0.80, −0.10), 0.004**	**−0.51 (−0.85, −0.16), 0.001**	−0.17 (−0.49, 0.16), 1.000	−0.06 (−0.47, 0.35), 1.000	0.28 (−0.10, 0.67), 0.317	−0.34 (−0.73, 0.05), 0.122
SVF performance	−1.81 (−4.17, 0.55), 0.252	**−4.65 (−7.00, −2.31), <0.001**	−1.89 (−4.07, 0.30), 0.135	**−2.84 (−5.62, −0.60), 0.042**	−0.07 (−2.71, 2.57), 1.000	**−2.77 (−5.42, −0.12), 0.036**
PVF performance	−1.19 (−2.82, 0.43), 0.309	**−2.17 (−3.81, −0.54), 0.003**	−0.93 (−2.45, 0.60), 0.642	−0.98 (−2.89, 0.93), 1.000	0.27 (−1.55, 2.08), 1.000	−1.24 (−3.08, 0.59), 0.431
Naming	**−1.47 (−2.57. -0.36), 0.003**	**−1.49 (−2.60, −0.39), 0.002**	−0.66 (1.68, 0.37), 0.549	−0.03 (−1.33, 1.28), 1.000	0.81 (−0.43, 2.05), 0.502	−0.84 (−2.09, 0.42), 0.462
Comprehension	−0.09 (−0.95, 0.78), 1.000	**−0.96 (−1.83, −0.08), 0.023**	−0.45 (−1.26, 0.36), 0.851	−0.87 (−1.88, 0.14), 0.136	−0.36 (−1.32, 0.60), 1.000	−0.51 (−1.48, 0.47), 1.000
Repetition	**−0.71 (−1.32, −0.09), 0.014**	**−0.67 (−1.28, −0.07), 0.021**	0.05 (−0.52, 0.61), 1.000	0.03 (−0.68, 0.75), 1.000	**0.75 (0.07, 1.43), 0.021**	**−0.72 (−1.40, −0.04), 0.032**

*Notes:* MCI: mild cognitive impairment; aMCI-SD: single-domain amnestic MCI; naMCI-SD: single-domain non-amnestic MCI; aMCI-MD: multi-domain amnestic MCI; naMCI-MD: multi-domain non-amnestic MCI; SVF: semantic verbal fluency; PVF: phonemic verbal fluency; BNT-sf: Boston Naming Test short form; Positive mean differences conventionally represent a superior performance of the first group of participants as quoted at the top of each column while negative mean differences correspond to a superior performance of the second group of participants as quoted at the top of each column; **bold** denotes statistical significance of the results.

## Discussion

In the present study we investigated language differences among older individuals with CN, MCI and AD focusing on the potential discrepancies between patients with amnestic and naMCI. Overall, according to our findings, participants could be hierarchically arranged in a hypothetical continuum of language decline, with CN individuals constituting the healthy reference and naMCI, aMCI, and AD patients representing gradually declining classes in terms of language performance. Although the direct comparison of amnestic vs naMCI was indicative of significant differences only in the case of SVF, language discrepancies were consistently less prominent between aMCI and AD participants (compared to naMCI and AD participants) as well as between CN and naMCI subjects (compared to aMCI and CN participants. The poorer performance of the aMCI group appeared to be mainly driven by the aMCI-MD subgroup, except for the SVF task in which patients with naMCI-SD outperformed those with aMCI-SD according to an effect size similar to the primary analysis (involving both single- and multi-domain amnestic and naMCI).

As mentioned before, previous research tends to be inconsistent regarding language differences (most notably with respect to verbal fluency measures) between amnestic and naMCI individuals. Evidence is more congruous, however, for individuals with aMCI-MD, consistently suggesting a poorer verbal fluency performance compared to those with single domain MCI (amnestic or non-amnestic), with several investigators proposing that aMCI-MD patients perform more like AD patients and less like individuals with single domain MCI (Brandt & Manning, 2009; Jokel, Seixas Lima, Fernandez, & Murphy, 2019; Weakley et al., 2013). These findings are also in accordance with the greater risk of dementia development (especially of the AD type) in the aMCI-MD group (Alexopoulos, Grimmer, Perneczky, Domes, & Kurz, 2006; Rasquin, Lodder, Visser, Lousberg, & Verhey, 2005; Tabert et al., 2006). On the other hand, to our knowledge, verbal fluency tasks have not been investigated previously via separation of single from multidomain naMCI. According to our findings, in terms of SVF, naMCI-MD patients performed better than older adults with aMCI-MD, similarly to those with aMCI-SD and worse than naMCI-SD individuals. On the other hand, in terms of PVF, subjects with naMCI-MD recorded a similar performance to those with single-domain amnestic and naMCI. Only those with aMCI-MD documented significantly lower PVF scores compared to individuals with naMCI-SD.

These findings appear to be in harmony with the very psychological construct of verbal fluency. Several cognitive skills have been suggested to contribute to the exquisitely complex functions of semantic and PVF. In specific, frontally mediated executive functions have been suggested to coordinate the retrieval of appropriate words, initiating word production and monitoring the whole process while inhibiting inappropriate and previously generated responses (Henry, Crawford, & Phillips, 2004). Moreover, fluency measures require access to the semantic-lexical stores, a function mediated by the temporal lobe (Lezak et al., 2004). In addition to the above pivotal functions, immediate attention and concentration are also important, as is the case in most cognitive assessments (Ruff, Light, Parker, & Levin, 1997). Although frontal pathology, executive dysfunction and attentional deficits have been associated with poorer performance in both fluency measures (Weakley et al., 2013), SVF decline has been additionally related to temporal lobe pathology (Hodges, Patterson, Oxbury, & Funnell, 1992). SVF is more heavily based on semantic stores, because its process involves the retrieval of appropriate exemplars fitting a specific semantic category, which has been long-suggested as the main reason of the poorer performance of patients with temporal lobe pathology (e.g., AD) on SVF tasks (Butters, Granholm, Salmon, Grant, & Wolfe, 1987). Of course, it is appropriate to acknowledge that the cognitive functions contributing to verbal fluency may be differentiated between normal and cognitively impaired individuals, adding to the already considerable complexity of these measures.

Considering the above, it appears logical that individuals with naMCI-SD outclassed those with aMCI in SVF. On the other hand, the potentially aggregating, more widespread neuropathology of the naMCI-MD group may account for the accumulation of additional cognitive impairment (e.g., executive deficits or attention deficits) that adversely affect SVF through courses irrelevant to the impairment of semantic storage, leading to an SVF performance that emulates that of the aMCI-SD group. Finally, given the aggregating, widespread neuropathology of the aMCI-MD group on the grounds of an impaired semantic reservoir, aMCI-MD patients unsurprisingly exhibited the worst performance on SVF. PVF scores were found to be relatively uniform among the MCI groups, with the exception of the aMCI-MD group that demonstrated a poorer performance than the naMCI group. Again, the combination of semantic (lexical repertoire) along with non-semantic cognitive dysfunction appears to afflict PVF performance more than isolated amnestic or non-amnestic, as well as multi domain non-amnestic dysfunction (although differences are less prominent that in the case of semantic fluency).

Published literature is substantially limited regarding confrontation naming, comprehension, and repetition in the patient subgroups that were the focus of the present study. Although confrontation naming fundamentally relies on the knowledge-semantic store, it also depends on intact attention and perceptual processing as well as activation of the appropriate semantic associations (Rohrer et al., 2007). In cases of mild AD, picture naming decline (non-response or false responses) has been mostly attributed conceptual degradation (difficulty in accessing the meaning of words) and impaired phonemic encoding (Moayedfar, Purmohammad, Shafa, Shafa, & Ghasisin, 2019) while high-frequency (commonly encountered) and early acquired objects tend to be more resistant to anomia (Cuetos, Rodríguez-Ferreiro, Sage, & Ellis, 2012). Verbal comprehension and repetition deficits usually coexist with word-finding difficulties since they likewise depend on semantic knowledge (especially verbal comprehension) and attention-perceptual functions (especially repetition) as well as the ability to coordinate verbal input and output pathways (Rohrer et al., 2007). Considering the importance of both semantic and non-semantic functions in these measures, multi-domain pathology would be anticipated to afflict individual performances to a greater extent, with semantic deficits assuming a more important role in verbal comprehension and confrontation naming, whereas perceptual processing deficits would be expected to play an equally major part in verbal repetition. The pattern of differences among the four MCI subtypes appears to reflect the aforementioned theoretical background, with the aMCI-MD group demonstrating the relatively worst performance in the naming and comprehension tasks and an equally poor performance with the naMCI-MD group in the repetition task.

Overall, in the present study, we have addressed a major gap in the literature on MCI, that is the investigation of language performance via separation of single from naMCI-MD. It is appropriate, however, to point out several limitations of our study. First, the diagnostic process did not include biological and imaging biomarkers (they were not systematically collected according to the study protocol); therefore, despite the expert consensus clinical diagnosis, misclassification bias may yet be present. Moreover, although the random selection process ensured satisfactory generalizability properties for our results in community-dwelling older adults, non-response bias (participation bias) cannot be ruled out with absolute certainty (Dardiotis et al., 2014). Furthermore, our analyses accounted for only three demographic parameters, namely age, sex, and education. Therefore, the latent impact of unaccounted confounders on the investigated associations cannot be eliminated. In addition to the above, language performance was assessed on its own and was not compared to other cognitive domains, especially executive function, and episodic memory. Finally, similar to any study on MCI, the very diagnostic procedure and subtyping of MCI partially relied on the same neuropsychological tests used to investigate the domain of language (Lopez, 2006).

## Conclusions

In the present study investigating language performance, individuals with MCI performed at a level between those with CN and AD while participants with naMCI outperformed those with aMCI, especially in semantic fluency and comprehension tasks. The discrepancies between amnestic and naMCI were mainly driven by the aMCI-MD subtype. Exploring language differences via separation of single from multi-domain naMCI has provided a potential explanation for the contradictory evidence of previous research. In specific, the unaccounted inclusion of participants with either single or multi-domain MCI in different proportions, may have been responsible for the generation of contradictory associations among previous studies.

## Funding

This work was supported by the following grants: Alzheimer's Association (grant number: IIRG-09-133014); ESPA-EU program Excellence Grant (ARISTEIA), which is co-funded by the European Social Fund and Greek National resources (grant number: 189 10276/8/9/2011); Ministry for Health and Social Solidarity (Greece) (grant number: DY2b/oik.51657/14.4.2009). The funders had no role in the design, analysis or writing of this article.

## Conflict of interest

None declared.

## References

[ref1] Alexopoulos, P., Grimmer, T., Perneczky, R., Domes, G., & Kurz, A. (2006). Progression to dementia in clinical subtypes of mild cognitive impairment. Dementia and Geriatric Cognitive Disorders, 22(1), 27–34. 10.1159/000093101. 16679762

[ref2] American Psychiatric Association (2000). Diagnostic and statistical manual of mental disorders (4th ed.). Washington, DC: American Psychiatric Association Press.

[ref3] Amunts, J., Camilleri, J. A., Eickhoff, S. B., Heim, S., & Weis, S. (2020). Executive functions predict verbal fluency scores in healthy participants. Scientific Reports, 10(1), 11141. 10.1038/s41598-020-65525-9. 32636406 PMC7341845

[ref4] Benton, A. L., Silvan, A. B., & Hamsher, K. (1994). Contributions to neuropsychological assessment: A clinical manual (2nd ed.). New York: Oxford University Press.

[ref5] Blanc, F. (2013). Does cognitive profile distinguish Lewy body disease from Alzheimer’s disease in the early stages? Journal of Neurology, Neurosurgery, and Psychiatry, 84(12), 1299–1300. 10.1136/jnnp-2012-304694. 23591555

[ref5a] Bombois, S., Debette, S., Bruandet, A., Delbeuck, X., Delmaire, C., Leys, D., & Pasquier, F. (2008). Vascular subcortical hyperintensities predict conversion to vascular and mixed dementia in MCI patients. Stroke, 39(7), 2046–2051. 18436882 10.1161/STROKEAHA.107.505206

[ref6] Bougea, A., Maraki, M. I., Yannakoulia, M., Stamelou, M., Xiromerisiou, G., Kosmidis, M. H., et al. (2019). Higher probability of prodromal Parkinson disease is related to lower cognitive performance. Neurology, 92(19), e2261–e2272. 10.1212/WNL.0000000000007453. 30944240

[ref7] Bozikas, V. P., Giazkoulidou, A., Hatzigeorgiadou, M., Karavatos, A., & Kosmidis, M. H. (2008). Do age and education contribute to performance on the clock drawing test? Normative data for the Greek population. Journal of Clinical and Experimental Neuropsychology, 30(2), 199–203. 10.1080/13803390701346113. 18938671

[ref8] Brandt, J., & Manning, K. J. (2009). Patterns of word-list generation in mild cognitive impairment and Alzheimer’s disease. The Clinical Neuropsychologist, 23(5), 870–879. 10.1080/13854040802585063. 19301196 PMC2857742

[ref9] Brouillette, R. M., Martin, C. K., Correa, J. B., Davis, A. B., Han, H., Johnson, W. D., et al. (2011). Memory for names test provides a useful confrontational naming task for aging and continuum of dementia. Journal of Alzheimer’s Disease: JAD, 23(4), 665–671. 10.3233/JAD-2011-101455. PMC3934750 21304184

[ref10] Butters, N., Granholm, E., Salmon, D. P., Grant, I., & Wolfe, J. (1987). Episodic and semantic memory: A comparison of amnesic and demented patients. Journal of Clinical and Experimental Neuropsychology, 9(5), 479–497. 10.1080/01688638708410764. 2959682

[ref11] Coutinho, A. M. N., Porto, F. H. G., Duran, F. L. S., Prando, S., Ono, C. R., Feitosa, E. A. A. F., et al. (2015). Brain metabolism and cerebrospinal fluid biomarkers profile of non-amnestic mild cognitive impairment in comparison to amnestic mild cognitive impairment and normal older subjects. Alzheimer’s Research & Therapy, 7(1), 58. 10.1186/s13195-015-0143-0. PMC4572657 26373380

[ref12] Csukly, G., Sirály, E., Fodor, Z., Horváth, A., Salacz, P., Hidasi, Z., et al. (2016). The differentiation of amnestic type MCI from the non-amnestic types by structural MRI. Frontiers in Aging Neuroscience, 8. 10.3389/fnagi.2016.00052. PMC4811920 27065855

[ref13] Cuetos, F., Rodríguez-Ferreiro, J., Sage, K., & Ellis, A. W. (2012). A fresh look at the predictors of naming accuracy and errors in Alzheimer's disease. Journal of Neuropsychology, 6(2), 242–256. 10.1111/j.1748-6653.2011.02025.x. 22284909

[ref14] Dalrymple-Alford, J. (2001). Comparative neuropsychology of Lewy body and Alzheimer’s dementia. Journal of Neurology, Neurosurgery, and Psychiatry, 70(2), 148. 10.1136/jnnp.70.2.148. 11160460 PMC1737200

[ref15] Dardiotis, E., Kosmidis, M. H., Yannakoulia, M., Hadjigeorgiou, G. M., & Scarmeas, N. (2014). The Hellenic longitudinal investigation of aging and diet (HELIAD): Rationale, study design, and cohort description. Neuroepidemiology, 43(1), 9–14. 10.1159/000362723. 24993387

[ref16] Dugger, B. N., Davis, K., Malek-Ahmadi, M., Hentz, J. G., Sandhu, S., Beach, T. G., et al. (2015). Neuropathological comparisons of amnestic and nonamnestic mild cognitive impairment. BMC Neurology, 15(1), 146. 10.1186/s12883-015-0403-4. 26289075 PMC4545878

[ref17] von Elm, E., Altman, D. G., Egger, M., Pocock, S. J., Gøtzsche, P. C., Vandenbroucke, J. P., et al. (2014). The strengthening the reporting of observational studies in epidemiology (STROBE) statement: Guidelines for reporting observational studies. International Journal of Surgery (London, England), 12(12), 1495–1499. 10.1016/j.ijsu.2014.07.013. 25046131

[ref18] Espinosa, A., Alegret, M., Valero, S., Vinyes-Junqué, G., Hernández, I., Mauleón, A., et al. (2013). A longitudinal follow-up of 550 mild cognitive impairment patients: Evidence for large conversion to dementia rates and detection of major risk factors involved. Journal of Alzheimer’s Disease: JAD, 34(3), 769–780. 10.3233/JAD-122002. 23271318

[ref19] Ferman, T. J., Smith, G. E., Kantarci, K., Boeve, B. F., Pankratz, V. S., Dickson, D. W., et al. (2013). Nonamnestic mild cognitive impairment progresses to dementia with Lewy bodies. Neurology, 81(23), 2032–2038. 10.1212/01.wnl.0000436942.55281.47. 24212390 PMC3854825

[ref20] Fischer, P., Jungwirth, S., Zehetmayer, S., Weissgram, S., Hoenigschnabl, S., Gelpi, E., et al. (2007). Conversion from subtypes of mild cognitive impairment to Alzheimer dementia. Neurology, 68(4), 288–291. 10.1212/01.wnl.0000252358.03285.9d. 17242334

[ref21] Folia, V., Liampas, I., Ntanasi, E., Yannakoulia, M., Sakka, P., Hadjigeorgiou, G., et al. (2022). Longitudinal trajectories and normative language standards in older adults with normal cognitive status. Neuropsychology, 36(7), 626–639. 10.1037/neu0000843. 35797176

[ref22] Folstein, M. F., Folstein, S. E., & McHugh, P. R. (1975). Mini-mental state. Journal of Psychiatric Research, 12(3), 189–198. 10.1016/0022-3956(75)90026-6. 1202204

[ref23] Forlenza, O. V., & Chiu, E. (2008). Mild cognitive impairment: A concept ready to move on? Current Opinion in Psychiatry, 21(6), 529–532. 10.1097/YCO.0b013e328316c2ab. 18852557

[ref24] Giaglis, G., Kyriazidou, S., Paraskevopoulou, E., Tascos, N., & Kosmidis, M. H. (2010). Evaluating premorbid level: Preliminary findings regarding the vulnerability of scores on cognitive measures in patients with MS. Journal of the International Neuropsychological Societyp,15(Suppl. 1), i–219.

[ref25] Hamilton, C. A., Matthews, F. E., Donaghy, P. C., Taylor, J.-P., O’Brien, J. T., Barnett, N., et al. (2021). Cognitive decline in mild cognitive impairment with Lewy bodies or Alzheimer disease: A prospective cohort study. The American Journal of Geriatric Psychiatry, 29(3), 272–284. 10.1016/j.jagp.2020.07.018. 32863138

[ref26] Henry, J. D., Crawford, J. R., & Phillips, L. H. (2004). Verbal fluency performance in dementia of the Alzheimer’s type: A meta-analysis. Neuropsychologia, 42(9), 1212–1222. 10.1016/j.neuropsychologia.2004.02.001. 15178173

[ref27] Hodges, J. R., Patterson, K., Oxbury, S., & Funnell, E. (1992). Semantic dementia: Progressive fluent aphasia with temporal lobe atrophy. Brain, 115(6), 1783–1806. 10.1093/brain/115.6.1783. 1486461

[ref28] Hodges, J. R., Salmon, D. P., & Butters, N. (1990). Differential impairment of semantic and episodic memory in Alzheimer’s and Huntington’s diseases: A controlled prospective study. Journal of Neurology, Neurosurgery, and Psychiatry, 53(12), 1089–1095. 10.1136/jnnp.53.12.1089. 2149861 PMC488322

[ref29] Hughes, T. F., Snitz, B. E., & Ganguli, M. (2011). Should mild cognitive impairment be subtyped? Current Opinion in Psychiatry, 24(3), 237–242. 10.1097/YCO.0b013e328344696b. 21346570 PMC3365571

[ref30] Jokel, R., Seixas Lima, B., Fernandez, A., & Murphy, K. J. (2019). Language in amnestic mild cognitive impairment and dementia of Alzheimer’s type: Quantitatively or qualitatively different? Dementia and Geriatric Cognitive Disorders Extra, 9(1), 136–151. 10.1159/000496824.

[ref31] Kavé, G., & Sapir-Yogev, S. (2020). Associations between memory and verbal fluency tasks. Journal of Communication Disorders, 83, 105968. 10.1016/j.jcomdis.2019.105968. 31835071

[ref32] Kendell, R., & Jablensky, A. (2003). Distinguishing between the validity and utility of psychiatric diagnoses. American Journal of Psychiatry, 160(1), 4–12. 10.1176/appi.ajp.160.1.4. PMID: 12505793. 12505793

[ref33] Kim, B. S., Kim, Y. B., & Kim, H. (2019). Discourse measures to differentiate between mild cognitive impairment and healthy aging. Frontiers in Aging Neuroscience, 11, 221. 10.3389/fnagi.2019.00221. 31507406 PMC6714864

[ref34] Kosmidis, M. H., Tsotsi, S., Karambela, O., Takou, E., & Vlahou, C. H. (2010). Cultural factors influencing performance on visuoperceptual neuropsychological tasks. Behavioural Neurology, 23(4), 245–247. 10.1155/2010/956709. 21422566 PMC5434400

[ref35] Kosmidis, M. H., Vlachos, G. S., Anastasiou, C. A., Yannakoulia, M., Dardiotis, E., Hadjigeorgiou, G., et al. (2018). Dementia prevalence in Greece: The Hellenic longitudinal investigation of aging and diet (HELIAD). Alzheimer Disease and Associated Disorders, 32(3), 232–239. 10.1097/WAD.0000000000000249. 29528855

[ref36] Kosmidis, M. H., Vlahou, C. H., Panagiotaki, P., & Kiosseoglou, G. (2004). The verbal fluency task in the Greek population: Normative data, and clustering and switching strategies. Journal of the International Neuropsychological Society: JINS, 10(2), 164–172. 10.1017/S1355617704102014. 15012836

[ref37] Lezak, M. D., Howieson, D. B., Loring, D. W., Hannay, H. J., & Fischer, J. S. (2004). Neuropsychological assessment (4th ed.). New York: Oxford University Press.

[ref38] Liampas, I., Folia, V., Ntanasi, E., Yannakoulia, M., Sakka, P., Hadjigeorgiou, G., et al. (2022). Longitudinal episodic memory trajectories in older adults with normal cognition. The Clinical Neuropsychologist, 1–18. 10.1080/13854046.2022.2059011. 35400289

[ref39] Lopez, O. L. (2006). Neuropsychological characteristics of mild cognitive impairment subgroups. Journal of Neurology, Neurosurgery & Psychiatry, 77(2), 159–165. 10.1136/jnnp.2004.045567. 16103044 PMC2077558

[ref40] McCullough, K. C., Bayles, K. A., & Bouldin, E. D. (2019). Language performance of individuals at risk for mild cognitive impairment. Journal of Speech, Language, and Hearing Research, 62(3), 706–722. 10.1044/2018_JSLHR-L-18-0232. 30950734

[ref41] McDonnell, M., Dill, L., Panos, S., Amano, S., Brown, W., Giurgius, S., et al. (2020). Verbal fluency as a screening tool for mild cognitive impairment. International Psychogeriatrics, 32(9), 1055–1062. 10.1017/S1041610219000644. 31258101 PMC9153280

[ref42] McKhann, G., Drachman, D., Folstein, M., Katzman, R., Price, D., & Stadlan, E. M. (1984). Clinical diagnosis of Alzheimer’s disease: Report of the NINCDS-ADRDA work group* under the auspices of Department of Health and Human Services Task Force on Alzheimer’s disease. Neurology, 34(7), 939–939. 10.1212/WNL.34.7.939. 6610841

[ref43] Moayedfar, S., Purmohammad, M., Shafa, N., Shafa, N., & Ghasisin, L. (2019). Analysis of naming processing stages in patients with mild Alzheimer. Applied Neuropsychology: Adult., 28(1), 107–116. 10.1080/23279095.2019.1599894. 31030561

[ref44] Nutterupham, K., Saykin, A., Rabin, L., Roth, R., Wishart, H., Pare, N., et al. (2008). Verbal fluency performance in amnestic MCI and older adults with cognitive complaints. Archives of Clinical Neuropsychology, 23(3), 229–241. 10.1016/j.acn.2008.01.005. 18339515 PMC2743541

[ref45] Oulhaj, A., Wilcock, G. K., Smith, A. D., & de Jager, C. A. (2009). Predicting the time of conversion to MCI in the elderly: Role of verbal expression and learning. Neurology, 73(18), 1436–1442. 10.1212/WNL.0b013e3181c0665f. 19794124 PMC2882176

[ref47] Peltz, C. B., Corrada, M. M., Berlau, D. J., & Kawas, C. H. (2011). Incidence of dementia in oldest-old with amnestic MCI and other cognitive impairments. Neurology, 77(21), 1906–1912. 10.1212/WNL.0b013e318238ee89. 22076544 PMC3233189

[ref48] Petersen, R. C. (2004). Mild cognitive impairment as a diagnostic entity. Journal of Internal Medicine, 256(3), 183–194. 10.1111/j.1365-2796.2004.01388.x. 15324362

[ref49] Qin, R., Li, M., Luo, R., Ye, Q., Luo, C., Chen, H., et al. (2020). The efficacy of gray matter atrophy and cognitive assessment in differentiation of aMCI and na MCI. Applied Neuropsychology: Adult, 1–7, 83–89. 10.1080/23279095.2019.1710509. 31945304

[ref50] Rasquin, S. M. C., Lodder, J., Visser, P. J., Lousberg, R., & Verhey, F. R. J. (2005). Predictive accuracy of MCI subtypes for Alzheimer’s disease and vascular dementia in subjects with mild cognitive impairment: A 2-year follow-up study. Dementia and Geriatric Cognitive Disorders, 19(2–3), 113–119. 10.1159/000082662. 15591801

[ref51] Rende, B., Ramsberger, G., & Miyake, A. (2002). Commonalities and differences in the working memory components underlying letter and category fluency tasks: A dual-task investigation. Neuropsychology, 16(3), 309–321. 10.1037//0894-4105.16.3.309. 12146678

[ref52] Rinehardt, E., Eichstaedt, K., Schinka, J. A., Loewenstein, D. A., Mattingly, M., Fils, J., et al. (2014). Verbal fluency patterns in mild cognitive impairment and Alzheimer’s disease. Dementia and Geriatric Cognitive Disorders, 38(1–2), 1–9. 10.1159/000355558. 24556750

[ref53] Rohrer, J. D., Knight, W. D., Warren, J. E., Fox, N. C., Rossor, M. N., & Warren, J. D. (2007). Word-finding difficulty: A clinical analysis of the progressive aphasias. Brain, 131(1), 8–38. 10.1093/brain/awm251. 17947337 PMC2373641

[ref54] Rojas, J., & Bettcher, B. (2016). Non-amnestic mild cognitive impairment. In B. Miller & B. Boeve (Eds), The Behavioral neurology of dementia (pp. 232–244). Cambridge: Cambridge University Press. doi:10.1017/9781139924771.017

[ref56] Ruff, R. M., Light, R. H., Parker, S. B., & Levin, H. S. (1997). The psychological construct of word fluency. Brain and Language, 57(3), 394–405. 10.1006/brln.1997.1755. 9126423

[ref57] Sadiq, D., Whitfield, T., Lee, L., Stevens, T., Costafreda, S., & Walker, Z. (2017). Prodromal dementia with Lewy bodies and prodromal Alzheimer’s disease: A comparison of the cognitive and clinical profiles. Journal of Alzheimer’s Disease: JAD, 58(2), 463–470. 10.3233/JAD-161089. 28453473

[ref58] Sherman, J. C., Henderson, C. R., Jr., Flynn, S., Gair, J. W., & Lust, B. (2021). Language decline characterizes amnestic mild cognitive impairment independent of cognitive decline. Journal of Speech, Language, and Hearing Research, 64(11), 4287–4307. 10.1044/2021_JSLHR-20-00503. 34699277

[ref59] Szatloczki, G., Hoffmann, I., Vincze, V., Kalman, J., & Pakaski, M. (2015). Speaking in Alzheimer’s disease, is that an early sign? Importance of changes in language abilities in Alzheimer’s disease. Frontiers in aging. Neuroscience, 7, Article 195. 10.3389/fnagi.2015.00195. PMC4611852 26539107

[ref60] Tabert, M. H., Manly, J. J., Liu, X., Pelton, G. H., Rosenblum, S., Jacobs, M., et al. (2006). Neuropsychological prediction of conversion to Alzheimer disease in patients with mild cognitive impairment. Archives of General Psychiatry, 63(8), 916. 10.1001/archpsyc.63.8.916. 16894068

[ref61] Teng, E., Leone-Friedman, J., Lee, G. J., Woo, S., Apostolova, L. G., Harrell, S., et al. (2013). Similar verbal fluency patterns in amnestic mild cognitive impairment and Alzheimer’s disease. Archives of Clinical Neuropsychology, 28(5), 400–410. 10.1093/arclin/act039. 23752677 PMC3711375

[ref62] Tsapkini, K., Vlahou, C. H., & Potagas, C. (2010). Adaptation and validation of standardized aphasia tests in different languages: Lessons from the Boston diagnostic aphasia examination-short form in Greek. Behavioural Neurology, 22(3–4), 111–119. 10.3233/ben-2009-0256. 20595743 PMC5434391

[ref63] Vaughan, R. M., Coen, R. F., Kenny, R., & Lawlor, B. A. (2018). Semantic and phonemic verbal fluency discrepancy in mild cognitive impairment: Potential predictor of progression to Alzheimer’s disease: Verbal fluency discrepancy in MCI. Journal of the American Geriatrics Society, 66(4), 755–759. 10.1111/jgs.15294. 29572820

[ref64] Vlachos, G. S., Kosmidis, M. H., Yannakoulia, M., Dardiotis, E., Hadjigeorgiou, G., Sakka, P., et al. (2020). Prevalence of mild cognitive impairment in the elderly population in Greece: Results from the HELIAD study. Alzheimer Disease and Associated Disorders, 34(2), 156–162. 10.1097/WAD.0000000000000361. 31913961

[ref65] Vlahou, C. H., & Kosmidis, M. H. (2002). The Greek Trail making test: Preliminary normative data for clinical and research use. Psychology: The Journal of the Hellenic Psychological Society, 9(3), 336–352. 10.12681/psy_hps.24068.

[ref66] Vlahou, C. H., Kosmidis, M. H., Dardagani, A., Tsotsi, S., Giannakou, M., Giazkoulidou, A., et al. (2013). Development of the Greek verbal learning test: Reliability, construct validity, and normative standards. Archives of Clinical Neuropsychology, 28(1), 52–64. 10.1093/arclin/acs099. 23179043

[ref67] Wakefield, S., McGeown, W., Shanks, M., & Venneri, A. (2014). Differentiating normal from pathological brain ageing using standard neuropsychological tests. Current Alzheimer Research, 11(8), 765–772. 10.2174/156720501108140910121631. 25212915

[ref68] Weakley, A., Schmitter-Edgecombe, M., & Anderson, J. (2013). Analysis of verbal fluency ability in amnestic and non-amnestic mild cognitive impairment. Archives of Clinical Neuropsychology, 28(7), 721–731. 10.1093/arclin/act058. 23917346 PMC3888195

[ref69] Yarnall, A. J., Rochester, L., & Burn, D. J. (2013). Mild cognitive impairment in Parkinson’s disease. Age and Ageing, 42(5), 567–576. 10.1093/ageing/aft085. 23868092

